# Field Homology in the Brain of Vertebrates

**DOI:** 10.3390/biology15030248

**Published:** 2026-01-29

**Authors:** Luis Puelles, Elena Garcia-Calero

**Affiliations:** 1 Departamento de Anatomía Humana y Psicobiología, Facultad de Medicina, Universidad de Murcia, Campus de Ciencias de la Salud, 30120 Murcia, Spain; puelles@um.es; 2 Instituto Murciano de Investigación Biomédica (IMIB), 30120 Murcia, Spain

**Keywords:** Bauplan, sameness, developmental fields, morphogenetic pattern

## Abstract

In this commentary, we discuss the concept of field homology, particularly as applied to the brain in comparative neuromorphology. We emphasize its roots in the topological properties of the Bauplan notion (organization plan) as well as in the still scarcely understood concept of developmental fields, and we criticize their modernly frequent erred substitution by the search of similarity of characters. We defend the logical causal connection of embryonic homology with adult homology, irrespective of the regulatory aspects of ontogeny.

## Simple Summary

The concept of field homology is an important notion in comparative anatomy and neuroscience (of interest to applied and clinical neuroscience in judging the validity of animal models of the human body and brain parts). It smooths over the morphologic hypotheses of homology referring to either embryonic or adult animal forms (the latter refer to the fundamental sameness of body or brain parts according to a common Bauplan and shared developmental/molecular mechanisms, usually comparable between vertebrate animal models and the human). It applies wherever manifestly homologous embryonic primordia produce in two species variant (non-similar) final derivatives. It also facilitates tracing homologies between different levels of complexity (genomic, embryonic, and adult). We emphasize that similarity of form or function is not required (and may be misleading in cases of homoplasy) in a hypothesis of homology either on embryonic or adult forms. Homologs usually have some variant aspects. Fundamental sameness results from the embryonic existence of regulative developmental fields, able to achieve a specific morphostatic fate via a variety of mechanistic routes, thanks to their intrinsic cohesivity, coherence, and diverse intercellular communication mechanisms. We conclude that field homology is a valuable heuristic recourse, particularly for the comparative evolutionary analysis of the complex brain structure, for which there is little fossil evidence.

## 1. Introduction

Field homology is a concept applied by comparative neuromorphologists to cases where neural parts compared as relatively undifferentiated embryonic primordia are clearly homologous according to a shared topologic position in the brain structural plan and gene expression patterns, but show differences at later stages in the final derivatives that develop in different species (strata, layers or nuclei, or even cell types); the corresponding differentiated brain territories are said to be field-homologous, understanding the variations observed as a lack of similarity. There apparently exist problems in the acceptance of this criterium, which is general and does not need previous acceptance of any particular brain model.

If it is postulated that a particular part of the diencephalon, such as the pretectum, which has (say) N derived nuclei in birds and N + 3 in mammals, is *field homologous*, voices may protest that too much or too little is said. The concept of field homology holds that the bird pretectum taken as a whole can be homologized with the mammalian pretectum taken as a whole, since the primordium develops out of the same *developmental field* in terms of relative position in the neural tube primordium and molecular causal mechanisms. The different number of derivatives represents a histogenetic variation implying a lack of strict histologic similarity, that does not alter the fundamental homology, which is based essentially on the topologically invariant position of the pretectum in the brain Bauplan, its invariant composition by distinct progenitor areas, and similar developmental genes active in its construction. The different number of nuclear derivatives is a variant aspect of pretectal histogenesis that can perhaps be explained as due to peculiarities of intercellular adhesion, for instance the emergence of transcriptomically modified neuronal types that cause, by their new combinatorial adhesive properties, changes in cell aggregation (i.e., different number of nuclei). According to Owen’s [1] classic definition, which is still valid today, homology [sameness] refers to the same organ in different animals, *irrespective* of similarity in structure or function. Of course, that sort of topologic sameness can be attributed since Darwin [2] to monophyletic hereditary aspects of the evolution of the species compared. Unfortunately, in the case of brains, we know little or nothing about the inner structure of ancestral brains, since they do not fossilize, so that the phylogenetic criterium for homology cannot be applied in practice (leaving aside endocasts, and comparative developmental/genetic reconstruction).

Those who think that the sentence above about the postulated *field homology* of pretectal domains that produce different numbers of nuclei is too assertive are possibly haunted by the idea that developmental characters cannot be directly correlated with adult anatomical structure, due to the complexities and variability of developmental mechanisms and the resulting regulative aspects. Therefore, homology relationships supposedly cannot be transferred from embryonic to adult levels of analysis [3,4,5,6,7]. It is contended that the homology statement might be true in the embryo but wrong in the adult, or vice versa. These critics apparently demand developmental *similarity* to grant adult *sameness*, though they do admit that the adults can be non-similar. We think that neither adult nor embryonic (or genomic) homologs need to be similar; they just need to affect the same field of the Bauplan. This argument seems contaminated by a confusion of homology with similarity (see Owen’s definition above).

On the other hand, those who think that too little is offered in the statement above would like to know exactly what sort of similarity is implied between the postulated homologous pretectal parts of the avian and mammalian diencephalon. These critics probably would desire more evidence on detailed similarity in identified neuronal populations, including chemoarchitecture, connections, and functions. They might agree on the apparent basic topological and developmental sameness relative to an underpinning Bauplan but may think that such homology is too vague to be significant, if the derived parts are not sufficiently similar, or the homology cannot be postulated more precisely. There is the perceived danger that field homology might be used too easily to homologize anything. Indeed, it is in the eye of the beholder whether he considers all vertebrates (i.e., animals sharing a fundamental Bauplan) to be wholly field homologous one to another down to a certain level of regionalization.

The idea of sameness introduced by Owen [1] is such a marvelous idea that it still stimulates us nearly 200 years afterwards. It is well worth every effort to explore its depth. Note that homologizing is an innate capacity of our minds. Neonates spontaneously learn to distinguish cats from dogs, just by looking at them, and we apparently owe the first taxonomy to Aristoteles. It pleases us to detect that different natural tokens are basically the *same form*, with minor irrelevant variants.

Similar form, structure, or function (though often also apparent in homologous forms) do not represent relevant criteria to establish homologies because they can easily change adaptatively (just compare the noses of elephants and whales). This also happens with brains. We must accordingly rely instead on the historical analysis of relevant causal invariant features of Bauplan component structures within the *phylogenetic* and *ontogenetic* landscapes, to discern plausible cases of topological and structural sameness. None of these two reference backgrounds can be evaded, particularly in the brain, where we lack sufficient fossil data [2,8,9,10,11,12,13]. Evolutionary and developmental morphostatic mechanisms underpin homology, that is, sameness [14,15,16,17]. This occurs irrespective of variation and adaptation in morphogenesis, form, and function (note we have consciously added a third member—morphogenesis—to the usual statement; see [18,19,20]).

Topologic invariance is a sort of sameness that appears in the scenario as a reflection of either internal or external order, opposing the chaotic forces of change. Internal order (conservatism) is a deep biological trend, with roots in nucleic acid and protein thermodynamics and associated cell molecular biology. It leads to continuously inherited true sameness (conservation of pattern and structure over evolutionary time irrespective of significant molecular change), eventual incorporation of novelty into the genome (assimilation) and, sometimes, atavic or recurrent evolution of characters. External order refers to the epigenetic external parameters of life, which occasionally are such that they constrain viable morphostatic solutions, leading to convergent similarity (analogy) of some evolving characters and functions. However, body plans are apparently caused and maintained mainly by internal genomic causes [21,22].

The existence of parallel and convergent similarity-generating evolutionary possibilities is the reason why simple, unadorned similarity of adult characters, particularly without topological and developmental corroborating contexts, is suspect in the study of true homology. That faulty approach has led in the past to many failures. Paradoxically, this does not impede that most modern work in comparative neurobiology concentrates in checking *similarities* of one sort or another, even when facing problematic topological contexts, perhaps under the implicit assumption that homoplasy may be relatively rare. An important question that remains open is how much of morphogenesis implies genetic parallelism or convergence, that is, how far can true embryonic homology be separated causally from embryonic homoplasy [4,6,23]. All developmental agents (genes, transcription factors, signaling molecules, etc.) change at least partially during the eons of evolutionary time, duplicating, and losing or gaining developmental (or adult) functions. Their joint actions may achieve sameness, with or without appreciable similarity (as in the cases of the elephant and whale noses), meaning these factors are also the motors of non-similarity, and, eventually, of emergent novel structure or function. Nevertheless, true homology based on the morphostatic Bauplan apparently persists across the flux of genetic and developmental microphenomena, bespeaking of a metastability of its developmental and genomic underpinnings.

In this scenario, we would like to defend again the usefulness of the field homology concept (see commentary on the same subject by Puelles and Medina 2002 [24]), as we understand it in the brain or elsewhere, since it may represent a meeting point for other concepts of homology. We still believe that sound homology hypotheses stand upon an implicit field homology. Analysis of the concepts of “field” and “character” suggests that they are comparable. “Fields” are less rigid concepts than “characters” since they refer to histogenetic fields within a Bauplan (i.e., are not freely chosen by the researcher). The difficulties sketched at the beginning will be addressed after elaborating this point.

## 2. The Bauplan

This concept is so basic and well established that we often forget that we are assuming its existence. Embodied alternatively by the concepts of ‘type’, ‘morphotype’, or ‘body plan’, the German notion of Bauplan (“construction plan”) underpins classic and modern taxonomy [8,25,26]. This concept was commented upon by Gould [27], Hall [18], and Panchen [28]. In the meantime, it has become clear that, at the level of the Bauplan, structure and invariant topology (internal order, as defined above; see Hall [18]) are predominant, as was defended by Kuhlenbeck [13,29,30], based on Jacobshagen [11,12] (1925, 1927). This definition contrasted with the phylogenetic one introduced by Gegenbaur [10]. Kuhlenbeck held that phylogenetic ancestor relationships are of considerable explanatory interest but are irrelevant in practice for deducing homology, since Bauplan-based homology technically underpins taxonomy, and, therefore, it implies circular reasoning to use taxonomy (phylogenetic relationships) for defining homologies. A softer version of this strong statement considers, in addition to the Bauplan, other variables that involve adaptive modifications [18]. It is in these aspects where phylogenetic hierarchy and related cladistic analysis become useful instruments.

When we study potential homology of any character, we assume the corresponding Bauplan. The assumption of given common aspects of development may be less present in our subconscious. Studying the brain of vertebrates, we assume the vertebrate neural tube developmental paradigm, as well as the adult brain Bauplan [31]. Both changed recently, as paradigms sometimes do. The prevalent brain models during the 20th century were the ontogenetic one of W. His [32], and the columnar/functional adult model of Herrick [33,34,35,36]. Herrick’s functional predictions for the forebrain, in particular, have entailed unsurmountable modern difficulties because of its evident inconsistency with genoarchitectonic and experimental embryological patterning data. Recent reformulations of the equally old neuromeric model of brain morphogenesis (first proposed, among others, by Orr [37], von Kupffer [38], and Ziehen [39]) have been presented as likely alternative models, notably the prosomeric model of Puelles and Rubenstein. The latter is consistent with molecular and causal developmental data (reviews in [31,40,41,42,43,44]). However, there is, as yet, no general consensus about this change in paradigm.

Baupläne are composed of fundamental structural components (Grundbestandteile in German). These usually are represented by vaguely defined invariant gross aspects of form or structure (like head, trunk, and limbs in a body) and their respective anatomic subdivisions (Formbestandteile). We have to cut arbitrarily through bones/articulations, muscles, and nerves to separate a limb or the head from the trunk. We thus conclude that, when we conceive fundamental elements of the Bauplan, we assume simultaneously more or less complex boundary relationships, which “connect” each element to the whole (‘connections’ were introduced by Geoffroy Saint-Hilaire [26]). *Mutatis mutandis*, the same can be said for any “anatomical character” that might be considered for comparative purposes as a smaller distinguishable element within the Bauplan. We thus realize that brain parts must necessarily be intermeshed with one another, unifying the body plan.

This brings us to the concept of morphological fields. Conceptually, as used in physics, a ‘field’ implies a complex phenomenon whose position (center) can be located in space at least approximately (i.e., topologically), and where internal communications regulate the coherence of its internal phenomena, whereas its more or less imprecise boundary relationships reflect secondary interactions with surrounding fields. Boundary phenomena represent parameters with regard to the system state variables operating inside the field. Neighboring fields characteristically may penetrate each other in various ways, a crucial aspect that language tends to obscure by the assignation of distinct names.

Fields are found everywhere in the universe, from the cosmos to atomic particles. There are various sorts of fields with different physical magnitudes, equilibrium states, or states of stability. The complexity and lifetime of field phenomena change depending on the rules of the larger field within which the fields operate.

As suggested by Puelles and Medina (2002) [24], biological entities are formed by particularly complex sorts of fields, characterized by the intervention of thousands of different molecules and the consequent functional adaptability due to the genome and the considerable capacity for intercellular communication. The Bauplan of a live animal reflects the repeated attainment by such animal phenomena of functional equilibrium with the environment, via their reproduction.

## 3. Developmental Fields

The concept of *developmental or embryonic fields* was originally introduced 100 years ago by Gurwitsch [45,46], Weiss [47,48], Waddington [49,50], and Child [51]; these fields implement the full regulatory potencies of the zygotic genome in a hierarchy of morpho- or histogenetic primordia, each working under local positional and genetic rules. Early development up to gastrulation possibly occurs within a unitary developmental field, wherein the head-tail and dorsoventral polarities and bilateral symmetry are established. The corresponding boundary field elements are represented by the extraembryonic tissues. During subsequent ontogeny, after patterning, regionalization and growth, the newly specified differential state of given embryonic parts causes a change in rules and the emergence of field subdivisions such as head and trunk fields at early neural plate stages. These fields become subdivided again at later stages (brain tagmata, proneuromeres, neuromeres, major dorsoventral domains, like the alar and basal plates, and finally individual progenitor microzones). Primary and secondary organizers (i.e., sources of morphogen signals) help establish the new fields in interaction with the detection capacity of the cells and their genome relative to the diverse signals available. New interfield boundaries are influenced by signaling morphogens or mechanisms of intercellular communication used for positional information. Differential regulation of proliferative patterns may change the number of cells at each place.

In the end, sets of such fields are orderly aligned along the antero-posterior (AP) and dorso-ventral (DV) axes of the embryo. Their intrinsic microprocesses are relatively independent from the surrounding fields, leading to the emergence of differential fates and boundary relationships. As suggested by Puelles and Medina (2002) [24], internal phenomena within a developmental field typically show cohesiveness (i.e., common proliferative, cell-adhesion, and cell-communication properties), as well as regulative capacity. Each embryonic field builds a given part of the global Bauplan or subdivides into smaller fields that do so. It has been shown experimentally that a field can be extirpated, causing complete elimination of its derivatives. However, a small rest of the field often can regenerate the whole field fate. The right half of a neuromere can regenerate and repattern the missing left half.

The differential properties of developmental fields emerge from the gene combinations activated among component cells (note that some genes may be shared by all the cells, while others are differentially expressed). The self-regulatory stability of the field (its capacity to achieve its normal fate in various ways, independently of alterations or of the number of initial cells) is driven by interacting intercellular signals and the genomic sensitivity to any altered states, acting under local rules to organize the expression of particular combinations of transcription factors at every part of the field. These will guide local development along a particular route. Missing cells or molecules are replaced by newly induced ones or are substituted by redundant ones present in the field (such field properties are sometimes described as indicating an *attractor* ontogenetic mechanism). Homeotic fate transformations of a field may result from mutational or experimental changes in the genetic code of the field that imitate the code of the alternative fate (Kauffman [52] in “The Origin of Order”).

The effects of organizers usually are not instructive but positional, due to a concentration gradient of a signal molecule or morphogen, so that the distance to the signal source delimits various neuroepithelial subdomains where the response to the signal is a different histogenetic fate, implying a differential read-out of the genome. Boundary effects may orient the polarity of the field as a whole within a larger territory, but the field (in fact, the positionally selected or activated part of its genome) decides what structure is formed inside it.

Some fields are able to respond correctly to signals that normally do not reach them. For instance, pieces of the isthmic organizer or beads carrying the FGF8 protein (the morphogen secreted by this organizer) can experimentally induce an ectopic cerebellum in the alar plate of any of the 12 rhombomeres, as well as in the two caudal diencephalic prosomeres, or in the rostral midbrain, but not in the hypothalamic/telencephalic or prethalamic forebrain, caudal midbrain, or the spinal cord [53,54,55,56,57,58,59,60,61]. Normally, a cerebellum is produced only in the two rostralmost rhombomeres (r0, r1). In experiments inducing a supernumerary cerebellum in diverse rhombomeres, it was found that sometimes the cerebellum was oriented correctly (like the normal one), but in other cases, it was specularly oriented (probably due to the proximity of the ectopic cerebellum to either the local rostral or caudal interrhombomeric boundaries). In all cases, the ectopic cerebellum was normally organized into cortical and nuclear derivatives [60].

The isthmic organizer that emerges early on in vertebrates between the forebrain and hindbrain tagmata illustrates precisely the concept of a secondary organizer that provides positional information, in this case for the midbrain and the rostral prepontine or isthmocerebellar hindbrain. There is apparently a cerebellum-repressing role of high *Otx2* expression in the midbrain, a favorable *Gbx2* expression in absence of *Hox* genes in r0-r1 (isthmo-cerebellum domain), and a varied set of *Hox* genes in the more caudal rhombomeres. Other similarly operating differential genetic landscapes may apply at other boundaries elsewhere that likewise display organizer properties (e.g., the mid-diencephalic zona limitans influencing the fates of both thalamus and prethalamus or the acroterminal rostral domain affecting the hypothalamus). Other secondary organizers have been discovered (i.e., the hemispheric hem producing WNT signals, the pallio-subpallial boundary producing anti-WNT, or the medial preoptic versus lateral preoptic boundary producing SHH; see Puelles [62]). Possibly, all interneuromeric borders display some sort of organizer properties. This variety of signal sources may explain the diversity of field properties and fates obtained.

According to these experimental observations, progressive genoarchitectonic differentiation and consequent histogenetic regionalization of the neural tube wall may well reflect the stepwise regionalization of the neural plate field into field subdivisions with more distinct properties (active boundaries and types of internal signals). Such a compartmenting mechanism may reach, at some places, the size of individual neurons. For instance, individual photoreceptor subtypes differentiated in the retina are known to signal to immediately surrounding immature cells, inhibiting the differentiation of identical cell types. This suggests a final ‘salt and pepper’ dynamic in cell type differentiation.

Evidence accrued so far suggests that the neuroepithelial AP and DV subdivisions, and the variety of transcriptomic combinations observed in the developing brain, are progressively refined in a largely shared pattern across vertebrates. Theoretically, once new levels of shared subdivisions emerge, they become part of the vertebrate neural Bauplan and allow postulation of the corresponding homologies [31]. Insofar as the obtained subdivisions are not shared by some species, one-to-one homology ceases to apply and we only can speak of field homology. This simply restricts the validity of the homology to the subdivision level that remains shared. A ‘field-homologous pretectum’ means that a shared primary morphogenetic pattern exists (we assert that the compared species share a standard pretectum territory in their forebrain), irrespective of the fact that some non-homologous pretectal nuclei secondarily develop (unless it can be demonstrated that the different *number* of nuclei does not imply they present *different cell types*, but merely a variant in the histic distribution of *the same cell types*).

Early pallial domains detected in the telencephalon of agnathan vertebrates [40,63,64] seem to underlie subsequently emerged mammalian medial, dorsal, lateral, and ventral pallial domains [65,66], and lead to ulteriorly evolved more complex cortical regional and areal subdivision models [67,68,69]. In contrast, areal cortical subdivisions in mammals are a significant source of variation-lack of similarity-between these taxa.

During brain histogenesis, the undifferentiated initial radial epithelial structure of the brain wall changes to a more complex and diversely molecularly differentiated three-dimensional neural wall structure [43,70].

The incipient mantle layer radial stratification process is rather variable among extant vertebrates, irrespective of the fact that it operates on comparable cell types and roughly comparable neurogenetic heterochrony across the different fields [71]. Anamniotes generally display less radial neuronal migration (affecting migration range, inside-out or outside-in stratification, and number and types of migrating neurons) than amniotes. Birds and mammals represent separate high points in this aspect. Interestingly, elasmobranchs and teleosts have separately evolved some unique superficial neuronal populations, which are difficult to homologize with tetrapod entities (see Burrill and Easter on the zebrafish prosencephalic visual nuclei [72]).

The limited range of mantle radial migration found in urodeles has been hypothesized to be atavic (i.e., a step back in evolution). Nevertheless, the massed-up periventricular neurons of urodeles have been found to include all hodological and functional neuronal varieties observed in different radial positions in other tetrapods. Radial expansion of the mantle layer is thus independent from advanced transcriptomic regionalization, even when it introduces novel *adaptive functions* of the local circuitry due to the differential radial positions of some cell types. Other evolutionarily variable peculiarities of mantle histogenesis are due to specific tangential neuronal migrations. In this case, neurons produced in one histogenetic field sort out of its boundaries and stabilize later within other fields. While some tangential translocations seem to be shared among all vertebrate lineages (e.g., the subpallial inhibitory neurons moving into the diverse pallial territories [73,74,75,76]), others show less wide comparative distribution (e.g., pontine and olivary hindbrain migrations). Apart from motoneuronal migrations, which sometimes fail to occur—as occurs with the facial motoneuronal migration in birds—another known instance of not universally shared tangential migration occurs out of the rhombic lip, apparently occurring only in amniotes. It remains to be seen whether the homologous neuronal types that do not migrate can be identified at their respective origins.

The conclusion accordingly turns up that, Bauplan-wise, the processes of areal regionalization (molecular and histogenetic) are largely conserved and reasonably well-delimited (particularly the earlier they occur in development), and, accordingly, they underpin an invariant topologic fundament for field homology referred to the brain Bauplan. For full operativity, the full radial histogenetic domain derived from a developmental field should be taken as a unit for comparisons (a roughly quadrangular tissular volume—possibly deformed morphogenetically due to differential growth—that ranges from ventricular to pial surfaces, and includes all its cellular derivatives, including tangentially migrated ones; elements known to have migrated *into* such a unit should be discounted). On the other hand, mantle histogenesis can be variable in various aspects, due to the complexity of intervening differentiative and migratory neuronal mechanisms that operate variously in different species. Cell adhesivity differences may underpin such variation.

Field homologous brain areas accordingly are conceived as molecularly characteristic and positionally invariant derivatives from the same neuroepithelial embryonic field or within a conserved Bauplan that nevertheless display some secondary histogenetic differences due to variable mantle histogenetic phenomena (e.g., number of nuclei derived from a particular stratum, or differences in the local cell migrations). They are topologically and causally the same (in molecular background) in different vertebrate species, even if they seem different, i.e., non-similar (as with elephant and whale noses). They have an experimentally testable causal shared background in developmental field regulative and boundary properties, as well as in fundamental patterns of molecularly guided cell type differentiation. In principle, these units do not postulate more than a general sort of sameness of the respective derivatives in different species. There are, as yet, few comparative regional brain studies performed cogently with modern transcriptomic study methods that can discern homology relationships between individual cell types (but see, e.g., Tosches’s et al. study of the reptilian telencephalic dorsal ventricular ridge [77]). Consideration of the homologies postulated so far in the brain of vertebrates suggests that there frequently exist various levels of similarity, particularly in chemoarchitectonic and connectivity properties. These instances should be evaluated with regards to their potential true topologic and causal sameness, since they may not depend directly on the Bauplan and may be adaptive or convergent in various ways [18]. This implies, at the most, analogy, rather than homology.

Moreover, analysis of transcriptomically identified neuronal types within an appropriately defined Bauplan or brain model [44] is also relevant when we consider the possibility of field homology. Variation may exist in relative population sizes, range of migration, and resulting final positions in the brain wall (for instance, the selectively *Nr4a2*-expressing claustrum homolog lies *subpially*, at the correct Bauplan site, in the avian lateral pallium, whereas the mammalian claustrum lies *deep* to the insular cortex at the identical topologic lateral pallium site [78,79]; they obviously are not similar, and may even have non-identical connections, but they seem to be homologous according to our criteria [80]. For all we know, the claustrum brain character may have been inherited monophyletically from an unknown pre-reptilian shared ancestor of birds and mammals (if not from some earlier anamniote ancestor). The phylogenetic criterium of homology cannot be applied in practice, because we do not have the needed ancestors at hand, and because the brain does not fossilize. However, the claustrum’s evolutionary origin can be deduced approximately by cladistic analysis of extant taxa, as long as we know *where to look for it* (telencephalic pallial Bauplan) and agree about *gene markers* expected to label it. We just found the avian claustrum homolog, which supposedly did not exist, by using field homology.

The radial dimension of the original developmental field is indicated, theoretically, by the radial glia cells giving support to the derived neurons within the local mantle. Alternatively, clonal analysis can determine the field of relevant progenitors and the overall time course of local neurogenesis and cell migration, or the specific cell types produced over time. In this way, it may be assessed whether the neurons of interest are really comparable, representing a true field homology. This suggested approach contrasts with many modern studies using single cell transcriptomic analysis, which seem mainly interested in *similar functions* rather than identifying precisely where and when the distinct cell types are produced. Cell-type homologies may be hidden by local migratory phenomena or ambiguous adhesive properties, thus eliminating clearcut similarity, but such analysis needs to be construed in any case upon a field homology that refers the studied elements to a specific Bauplan or brain model [44].

In order to advance towards a solid field homology conclusion as we already recommended previously [24] (Puelles and Medina, 2002), the rule of thumb is to only compare elements that occupy the *same* (i.e., topologically equivalent) *place in the Bauplan*. This is importantly qualified by the developmental consideration that the characters to compare should not be chosen arbitrarily, since the significant cohesive and unitary subdivisions of the Bauplan are inescapably earmarked by the dynamics and temporal course of developmental field formation and subdivision during morphogenesis. The details of this process are only partly known and should be described as soon as possible. Only whole developmental fields and their respective ‘connections’ are sufficiently morphostatic.

## 4. The Difficulty of Insufficient Detail

Let us consider one of the difficulties of field homology mentioned in the Introduction, namely that it is too vague. We underline instead that the field homology concept allows more precise homologizing than is habitually the case. In the first place, the analysis starts by using developmental materials that corroborate a shared Bauplan; this exploits, to the full extent, their information content, without excluding ulterior detailed study of the chosen characters also in adult forms.

In practice, we first have to identify the relevant field candidate(s) consistently with the best modern morphological paradigm (model) and the Bauplan. For instance, if you choose the isthmus, then you also should look at the cerebellum, because they develop in the same field; there are reasons to suppose that each part somehow affects the development of the other. We need to identify the whole relevant field somehow (there exist nowadays several technical options based on distinct molecular profiles). As previously commented by Puelles and Medina (2002) [24], often, pure morphology does not help much (classic ventricular sulci or surface bulges in the brain wall are, by now, notorious wrong steppingstones); neuromeric boundaries may not be detectable in slowly proliferating species or at late developmental stages, but cryptic neuromeres can be detected molecularly, in so far as they are developmental fields [42]. Well-chosen molecular markers (RNA molecules, structural, membrane-located, or adhesive proteins, etc.) usually help to identify the developmental field in the predicted place. Fate maps also can be of help, though care must be taken to check the morphological assumptions under which they were constructed. Mantle layer histogenesis within the identified field can next be followed step by step by appropriate histological and molecular markers. The need to compare various section planes derives from the bending of the brain’s length axis; the theoretical and practical *relative topological positions* of interest need to be deduced from cross-correlated data in different section planes (e.g., the hypothalamus is not the floor of the diencephalon, as was classically wrongly assumed, but represents the rostral end of the neural tube [42,44]). We recommend examining the local orientation/disposition of radial glia processes and the relative positions of the corresponding ventricular and pial surfaces (these data are complementary to fate maps). The neurons in the brain do not float in a void, nor assemble into disordered nuclear masses as ‘potatoes in a potato sack’, a self-explanatory expression that was much appreciated by R. Nieuwenhuys, now sadly deceased [44]. The potatoes in our brains are always in remarkably specific locations and interconnect in remarkably specific ways. The fact that they are usually composed of varied types of neurons should not lead us to the wrong conclusion that they are chaotic. Thus, a formless sack of diverse sorts of neurons, devoid of radial references to their primary or secondary topologic ventricular and pial position in the brain wall, does not bring us closer to the proper Bauplan image of the brain whose structure we want to homologize. This needed precise field-related morphologic analysis needs someone, preferably human (rather than AI), that looks intelligently at the brain of interest. This allows us to learn which are the relevant spatial directions in the developmental field, or the directions in which signal gradients are diffusing or originally diffused. We recently found that the optimal sectioning plane for the mouse amygdala only could be chosen after several essays [81].

We would thus check that the developmental field of interest exists with a comparable molecular marker and boundary profile. This is usually the case when the field was correctly defined in one of the species compared, leaving aside differences in relative size. At this point, we examine relevant fate maps, radial glia, neurogenetic data, connections, other experimental results, etc.

If this analysis uncovers examples of parallel or convergent homoplasy, we should fall back on the closest (larger) field homology possible, which then itself becomes a new reference for taxonomic and cladistic analysis. All the effort implied is therefore scientifically worthwhile.

## 5. From Genes to Embryos to Adults

The other difficulty mentioned in the Introduction is the issue of whether different levels of homology, such as adult, embryonic, and genetic homologies, can be connected smoothly one to another. This point was analyzed by Striedter and Northcutt [6] and Striedter [4,5,7], who think these levels need to be considered separately, because the transitions between these levels usually involve a *lack of similarity* in the relevant mechanisms. Different individual genes intervene in the same developmental process in different species, and different developmental mechanisms sometimes conduce to the same adult organ. However, most authors in the comparative neuroanatomic field do not oppose the idea of the consistency of embryonic parts with adult parts, possibly due to the pervasive and well demonstrable existence of a widely conserved Bauplan. If everything changed with time both in ontogeny and phylogeny, to the point of disrupting the inherited homologies, how is it that we recognize uniformly a conserved Bauplan across millions of years? Do we have to understand, then, that specific genomic mechanisms do not reproducibly cause given embryonic primordia (both within a species and in diverse species), and embryonic primordia do not transform reproducibly into specific adult derivatives?

We feel that this conclusion of Northcutt and Striedter demands *strict similarity* at the levels of developmental mechanisms and genetic functions, something not required at the level of adult homologies (if they agree with Owen [1]). In practice, these authors seem to forget the important buffering role of genetic and developmental regulatory mechanisms involved in morphostasis. Their belief in the non-reducibility of adult homology into developmental and genetic homologies questions developmental and molecular analysis in comparative studies, a result that we think these authors do not endorse themselves [7], but others may well do so [23].

This preoccupation with the variability of genetic and embryologic mechanisms in contrast to the evidence of Bauplan morphostasis is nothing compared with a similar contemplation of a reduction in genomic molecular aspects to physical causes. The deterministic physical underpinnings of any living organism vary continuously at rates that well exceed the Brownian motions of molecular particles in cells, but genomes nevertheless conserve important genetic sequences for millions of years. Homology postulates conceived initially for taxonomy purposes on adult animal specimens already contemplated variation in form and function as irrelevant [1], and this reasonable consideration should not be negated at embryonic or genomic levels. We are interested in explaining onto- and phylogenetically, as well as molecularly, the objective *morphostatic aspects* of the construction plan of animals and plants.

It can be proposed that developmental fields are localized multicellular complexes of developing organs that *systematically* achieve the tissular fate that corresponds to their relative position in the full living organism *irrespective* of given redundancies and variations (and also of some experimental manipulations) in the molecular instruments and reactions occurring in the process and sometimes also in the cell types involved. Their limit lies in what the genome and the natural environment allow via the process of natural selection. They represent self-regulatory, biochemically driven cellular systems *selected* during millions of years to be able to insure with high probability the production of a particular functioning part of the brain or of the body under a specific Bauplan. Billions of years of an entire planet were needed to make them possible, while energy quanta and atomic particles were apparently available from the beginning of the universe. Homology deals with identifying, categorizing, and comparing these ontogenetic components of morphostatic living forms, registering when emergent variant properties arise, or when homoplasy occurs.

Adult homology indubitably exists (even if possibly in less cases than tradition may have conceived; see Striedter’s [4] comments on the abundance of convergent and parallel homoplasy). Ontogenesis and evolving genetic regulatory mechanisms necessarily underpin adult homology. How rich and variable these mechanisms are is irrelevant; we want to know what makes morphostasis stand out in the midst of evolution [82]. This means we have to keep our minds open to previously unforeseen theoretic solutions. Otherwise, we block the possibility to find them and carelessly may slip into giving, again, primacy to biological function over causal genetics and morphology. We also should examine critically the logic behind the published arguments for genetic and morphogenetic variability. These rationales may seem unimpeachable at first sight, though some of the evidence or the assumptions adduced might be seen under a different light and objected to. The zygote with its genome and appropriate epigenetic parameters causes embryonic development, and this eventually causes the functioning adult form, if not detoured accidentally into malformation or death. The only problem is in understanding *how* this happens, but it obviously happens. The concept of developmental fields presented above may allow us to look at the problem in a different light.

Puelles and Medina (2002) [24] already commented that “one of the problematic features we notice in the habitual treatment of embryonic homology is that the embryo is conceived as a small adult”. Since embryos obviously tend to have much the same Bauplan as the adult, it is usually forgotten that embryonic primordia belong to larger, as yet undivided, developmental fields. These will not be regulating exclusively the “character” in question, but probably several others besides it. The changing field properties over time may explain the stability of the morphogenetic result in spite of perturbations and different starting points of the values in the system variables (i.e., the case of the Wolffian lens). Such embryonic dynamics logically subside as the organism matures into a stable and functional adult form, maybe persisting only in homeostatic state control mechanisms.

The difficulties sketched at the beginning can then be resolved by a changing multiplicity of strictly constrained and regulated field-like transient developmental entities that work collectively in a highly ordered fashion (thus the Bauplan) to produce the full organism. Many of the molecules involved—particularly the transcription factors and the morphogens—function by “regulating regulators”, instead of as direct effectors of final structure. As explained by Ashby (1964) in An Introduction to Cybernetics [83], regulators typically block external variations from changing the internal state variables of the system (if the room thermostat works properly, there are no fluctuations in temperature, irrespective of external changes; the working of the regulator eliminates any mystery about why you obtain the same temperature with different initial states). Several chained gene networks control, as regulators, the embryonic state variables of developmental fields, thus increasing the epigenetic resilience of the system (i.e., independence from perturbance). The sensitivity of the primary regulator may be regulated by a secondary one, and the amplitude of its output signal may be regulated by yet another, etc.

The temporal course of evolution correlates with periods of early explosive variation and relatively rapid selection of Baupläne, followed by less marked or slower Bauplan modification with lower probability of new Baupläne afterwards. We now understand this relates to the emergence of complex regulation of multicellular interactions [21,27]. True evolutionary innovation involves cases where potentially possible novel equilibrium states of the system are explored within the pre-existent developmental fields. Their tendency to buffer perturbations (also of the novel obtainable equilibrium state) occasionally may tilt the original low probability of new positive uses for a variant of an already functioning molecular complex, some of whose elements may be recombined to novel positions in the causal flow chart. Thus, novel biochemical functions, and new cell types or structures, may emerge without distortion of the pre-existent overall equilibrium that insures Bauplan relationships, that is, conserving homology properties.

## 6. Conclusions

In conclusion, the evolutionary invention of molecular regulators underpins the stability and further increase in complexity of biological reactions taking place within the *developmental fields*. These are the only relevant embryonic “characters”. As ontogenesis advances, the available characters or fields become more regionalized and specific (compartmented). Developmental fields easily suggest the proposal of a hypothesis of field homology, limited exclusively in range and precision to the determination of the set of all its derivatives in the compared taxa. Since there is a temporal hierarchy of developmental fields, there must also be a hierarchy of field homologies that may be considered (there are limb homologies and finger homologies; are fingerprints homologous though not similar?).

Homology analysis may be pursued into the subdivisions of a developmental field. One important example that can be already distinguished relates to areal and regional subdivisions in the pallial cortex of mammals (see [69]). More evolved mammals have more differentiated areas in their cortex, but we can be sure that this leads to no chaotic loss of order and functionality and is faithfully reproduced in all normal tokens of a species. Cortical areas typically may represent the result of terminal developmental fields that are derivatives of earlier more encompassing (and more primitive) cortical developmental fields. It will need to be examined, following the pioneering work of Brodmann [84], how far these areal phenomena are the same across different mammals or can be compared to apparently less differentiated pallial regionalization phenomena detected in other vertebrate lineages. So far, very little use has been made of the molecular profile of different cortical areas. This approach is perfectly feasible now [69]. The field of cortical studies has spent the last 40 years largely examining which molecules and cell types *are general* across the entire cortex (see Yao et al. [85]), rather than investigating *what is different* in the functionally diverse areas that we know exist.

The corollary of this essay in field homology is not that field homology is an additional sort of homology notion to be used alternatively, side by side with the conventional phylogenetic and pre-evolutionary morphologic homology notions. Field homology is rather held to be *necessary* and, as already concluded by Puelles and Medina (2002) [24], “the only plausible fundament and methodology for integrating meaningfully historical, topological, biological and molecular homology causal approaches”. Care must be taken to distinguish homology from homoplasy, be it parallel or convergent.

## Data Availability

No new data were created or analyzed in this study. Data sharing is not applicable to this article.

## Glossary

*Field*: In the brain primordium, a field is a portion of the neuroepithelial wall that can be delimited by its distinct molecular profile in coincidence with available morphogenetic boundaries (e.g., transverse or longitudinal boundaries, vesicular stalks, etc.) and experimentally demonstrated specific fate.

*Developmental field*: Early field composed mainly of progenitor ventricular or neuroepithelial cells.

*Histogenetic field*: More advanced embryonic field where neurogenesis and gliogenesis are active and the mantle zone shows signs of histologic differentiation (strata, layers, nuclear primordia, developing tracts, and signs of tangential migration).

*Character*: Detail of organic structure chosen for comparisons across species in taxonomic cladistic analyses. We suggest they optimally should coincide with developmental or histogenetic fields.

*Bauplan*: Plan of organization of a body (body plan) or organ. It is a schematic ontological instrument that lists the main components of a complex structure, as well as any relevant (constant) subdivisions, together with a summary of their mutual spatial relationships relative to the main relevant spatial axes or dimensions.

*Neuromere*: Serial segmental or transverse subdivision of the neural tube of vertebrates. It is the smallest complete subdivision, of which there are 20 in the encephalic part of the brain, 7 in the forebrain, and 12 in the hindbrain. The neuromeres have the property of being metameric, which means they share a common internal organization schema, namely the division into dorsoventral longitudinal zones (floor, basal, alar, and roof plates).
